# Supplementary material for: PspA-mediated aggregation protects Streptococcus pneumoniae against desiccation on fomites
Source: mBio. 2023 Nov 20;14(6):e02634-23. doi: 10.1128/mbio.02634-23 (PMC10746202; doi:10.1128/mbio.02634-23)
Supplement: Supplemental Figures — Fig. S1 to S6. [file mbio.02634-23-s0001.pdf]

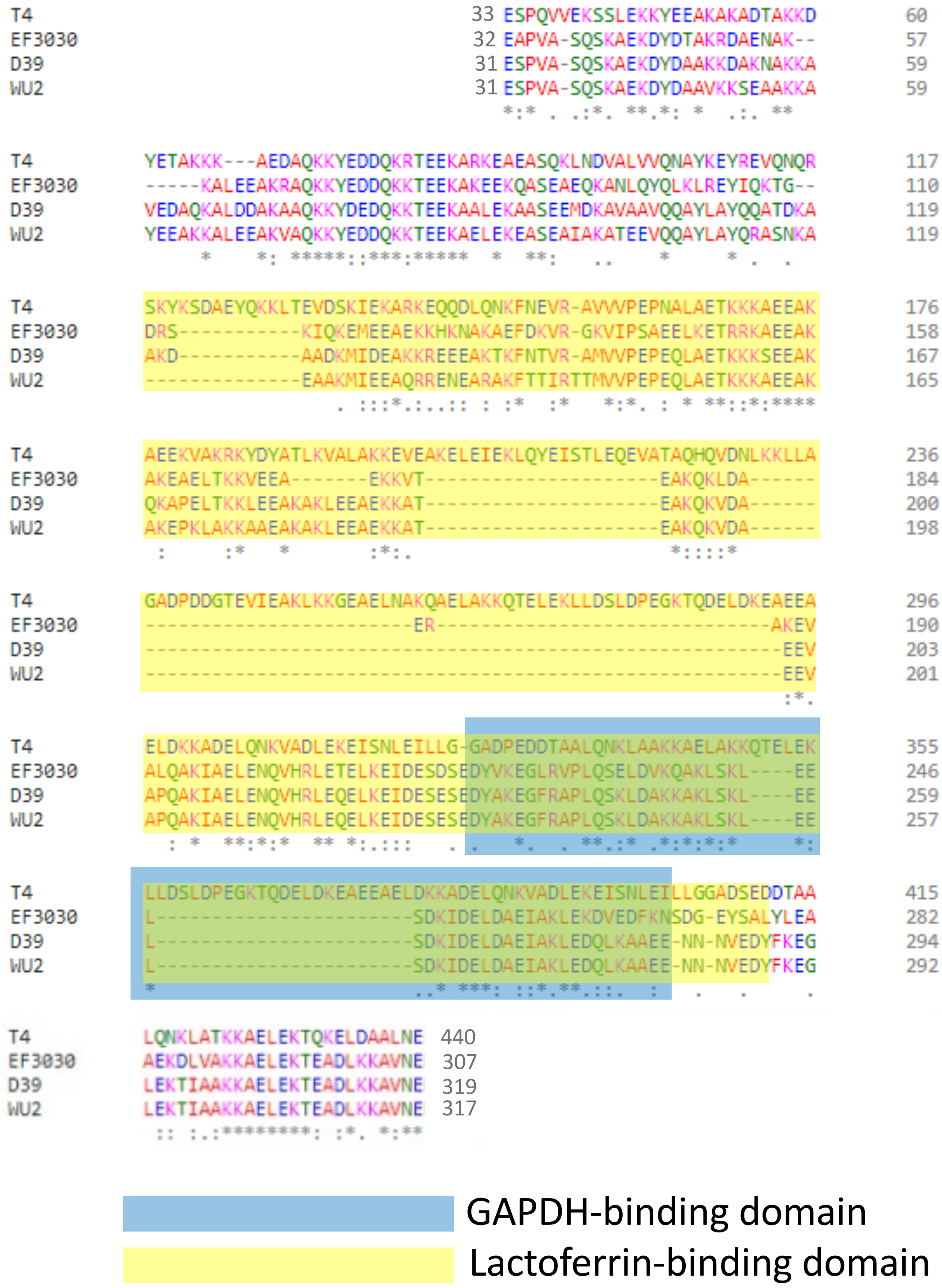

FIG S1 GAPDH and LF-binding domains of PspA across different *Spn* strains. Multiple sequence alignment of amino acid sequences of PspA for TIGR4 (serotype 4), EF3030 (serotype 19F), D39 (serotype 2), and WU2 (serotype 3). GAPDH-binding domain (blue) and LF-binding domain (yellow) are highlighted within the α-helical region.

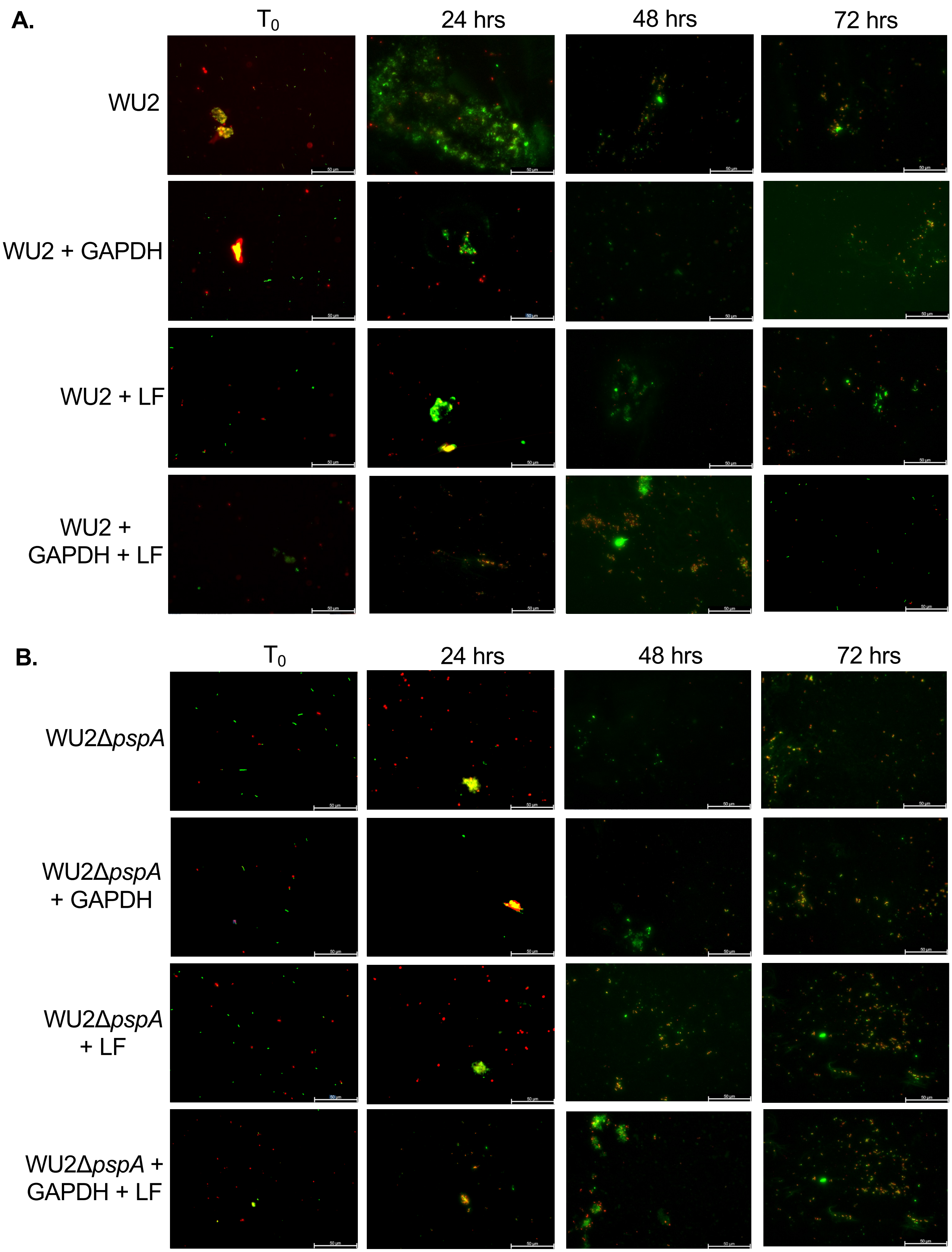

FIG S2 PspA-GAPDH complex protects *Spn* from desiccation *in vitro* over time. (A) WU2 and (B) WU2Δ*pspA* both in suspension (T_0) and desiccated were stained with SYTO 9 (green) and propidium iodide (red). Representative images for time “zero” (T_0) aggregated *Spn* and 24-, 48-, and 72-hours post desiccation *Spn* were taken at 10X magnification (scale bar=50 μm).

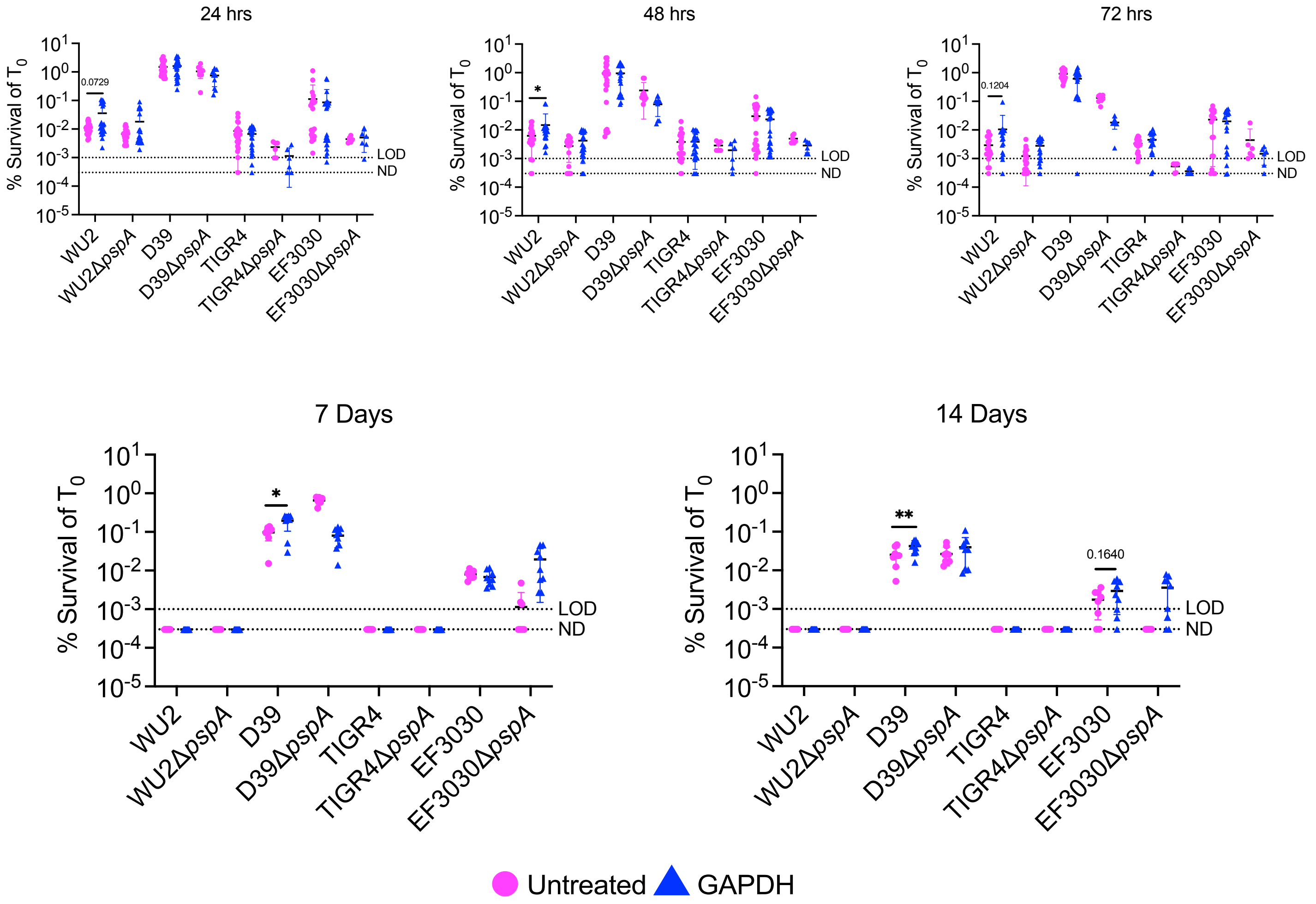

FIG S3 In vitro desiccation protection over time with multiple *Spn* strains. *Spn* strains WU2 (serotype 3), D39 (serotype 2), TIGR4 (serotype 4), EF3030 (serotype 19F), and their corresponding Δ*pspA* mutants were incubated with mGAPDH (10 µg/mL) and plated onto glass slides and allowed to dry for 24-, 48-, and 72-hours, 7 days, and 14 days. Bacterial survival was enumerated by CFUs based on T_0 survival. N=9-24 with the standard deviation (SD) shown. LOD=10^{-3} % survival. ND = 3 x 10^{-4} % survival. * = p ≤ 0.0332; ** = p ≤ 0.002.

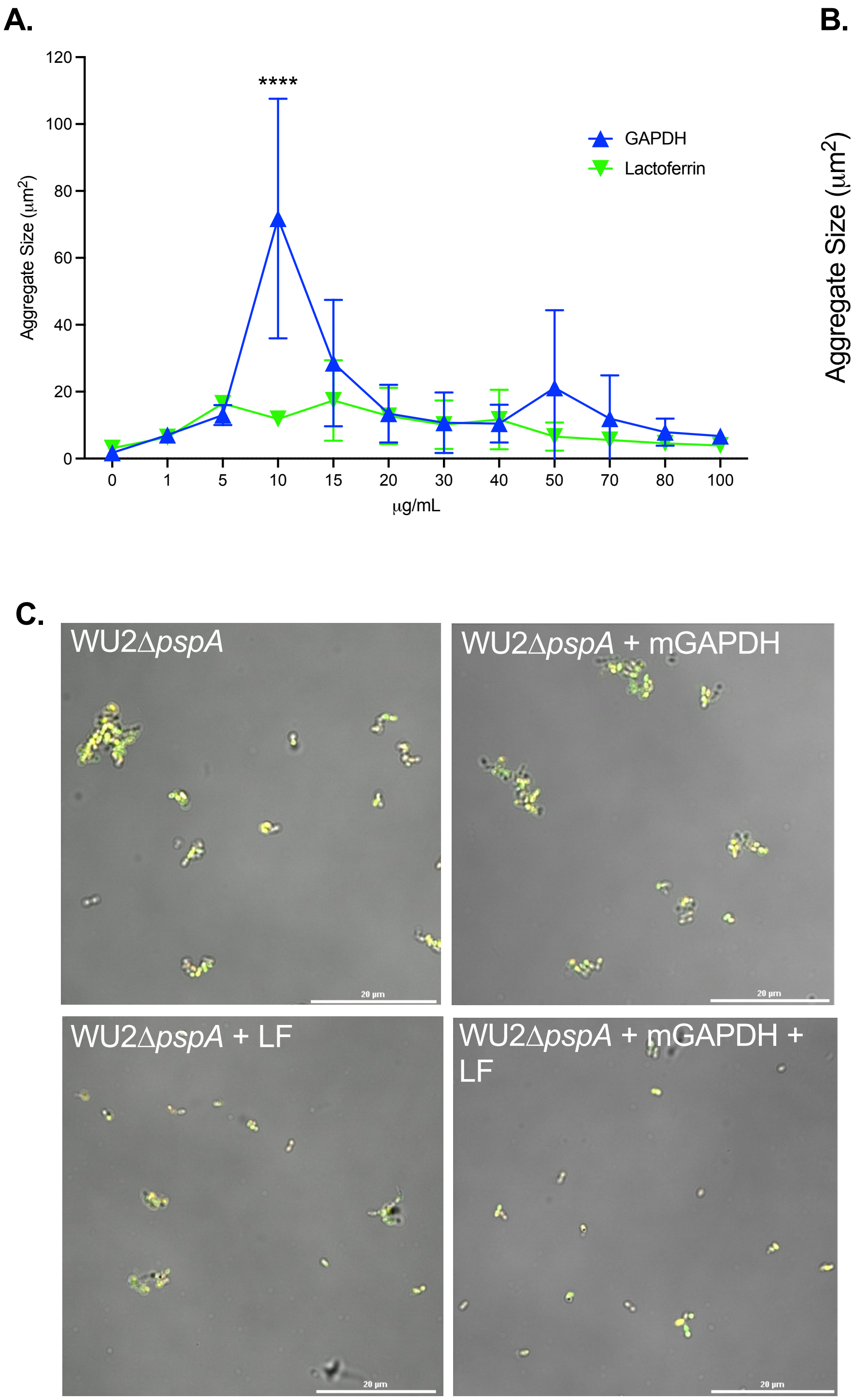

FIG S4 *Spn* is aggregated by GAPDH in a PspA-dependent manner. (A) Mean cluster size of the five largest clusters of WU2 per treatment with various concentrations of mGAPDH or LF (0-100 µg/mL) quantified from live bacteria. N=3 with the standard deviation (SD) shown (see methods for more details). (B) WU2 was incubated in solution with mGAPDH (10 µg/mL) over time. Mean cluster size of the five largest clusters of WU2 per timepoint was quantified from live bacteria. N=3 with the standard deviation (SD) shown (see methods for more details). (C) High resolution image of WU2Δ*pspA* pneumococci stained with SYTO 9 (green) and propidium iodide (red) and fixed with 4% paraformaldehyde and Fluoromount™ (see methods for more details). WU2Δ*pspA* shown when incubated in solution with mGAPDH, LF, or mGAPDH and LF (10 µg/mL) . All images captured at 60X magnification under oil immersion with a 20 µm scale bar (see methods for more details). * = $p \leq 0.0332$; **** = $p \leq 0.0001$.

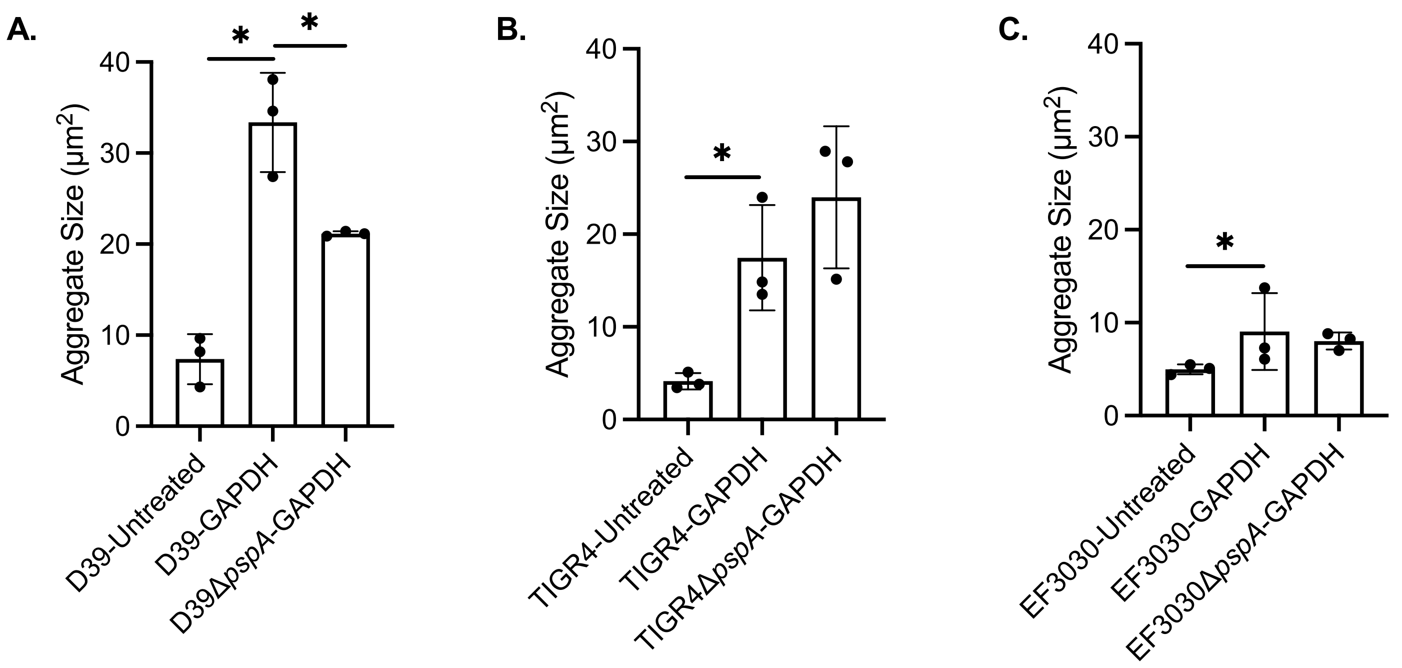

FIG S5 *Spn* is aggregated by GAPDH in a strain-dependent manner. *Spn* strains (A) D39 (serotype 2), (B) TIGR4 (serotype 4), (C) EF3030 (serotype 19F), and their corresponding Δ*pspA* mutants were stained with SYTO 9 (green) and propidium iodide (red) after incubation with and without mGAPDH (10 µg/mL) (see methods for more details). Mean cluster size of the five largest clusters per treatment (10 µg/mL) were then quantified from live bacteria (see methods for more details). N=3 with the standard deviation (SD) shown. * = p ≤ 0.0332.

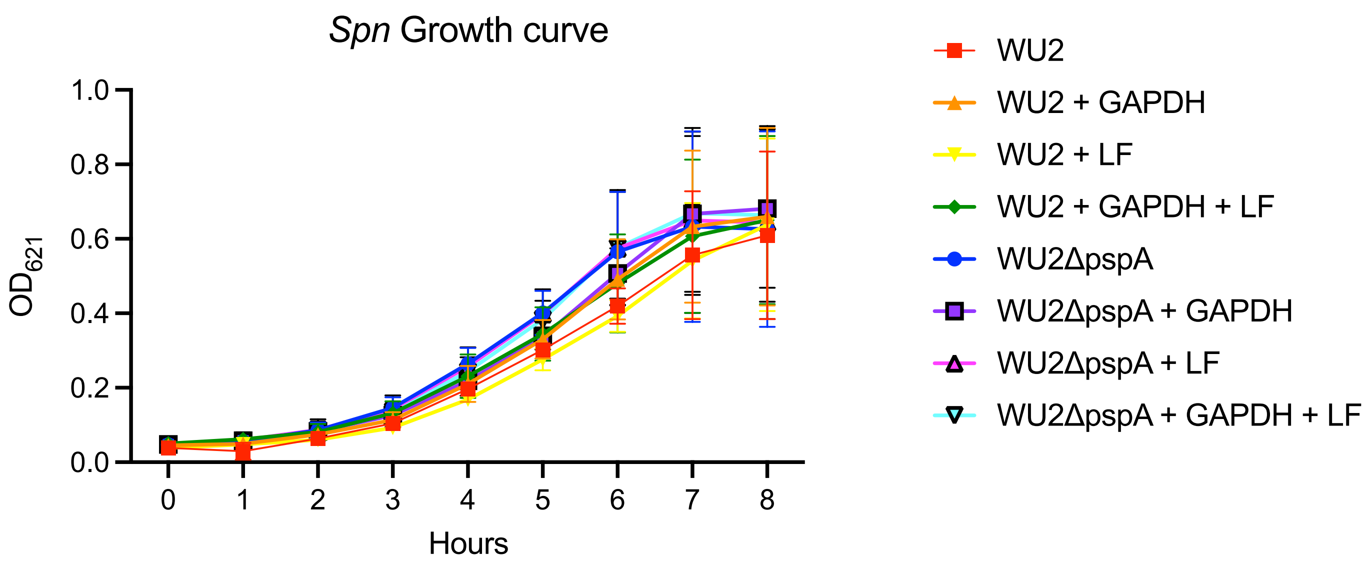

FIG S6 *Spn* growth is not affected by addition of GAPDH or LF. WU2 and WU2Δ*pspA* were grown in Todd Hewitt-Yeast (THY) broth with or without the addition of mGAPDH, LF, or with mGAPDH and LF (10 μg/mL) in an incubator at 37℃ with 5% CO_2 over 8 hours. OD_{621} was measured at each hour to record growth. N=3 with the standard deviation (SD) shown.
