# Supplementary material for: PspA-mediated aggregation protects Streptococcus pneumoniae against desiccation on fomites
Source: mBio. 2023 Nov 20;14(6):e02634-23. doi: 10.1128/mbio.02634-23 (PMC10746202; doi:10.1128/mbio.02634-23)
Supplement: Table S1 — Recovery of pneumococci on fomites following desiccation. [file mbio.02634-23-s0002.docx]

**Table S1. Recovery of pneumococci on fomites following desiccation (n=24-29/cohort).**

| **24 Hours** | **Recovered** | **Unrecoverable** | **% Recovered** | **P-value (Chi^2^)** |
| --- | --- | --- | --- | --- |
| **Untreated** |  | | | |
| WU2 | 29 | 0 | 100 | 0.0056 |
| WU2Δ*pspA* | 16 | 5 | 76 |  |
| **GAPDH** |  | | | |
| WU2 | 29 | 0 | 100 | NA |
| WU2Δ*pspA* | 21 | 0 | 100 |  |
| **Lactoferrin** |  | | | |
| WU2 | 24 | 0 | 100 | 0.1486 |
| WU2Δ*pspA* | 22 | 2 | 92 |  |
| **G + LF** |  | | | |
| WU2 | 24 | 0 | 100 | 0.3122 |
| WU2Δ*pspA* | 23 | 1 | 96 |  |

| **48 Hours** | **Recovered** | **Unrecoverable** | **% Recovered** | **P-value (Chi^2^)** |
| --- | --- | --- | --- | --- |
| **Untreated** |  | | | |
| WU2 | 12 | 16 | 43 | 0.3046 |
| WU2Δ*pspA* | 6 | 15 | 29 |  |
| **GAPDH** |  | | | |
| WU2 | 28 | 0 | 100 | 0.0004 |
| WU2Δ*pspA* | 13 | 8 | 62 |  |
| **Lactoferrin** |  | | | |
| WU2 | 14 | 10 | 58 | 0.0417 |
| WU2Δ*pspA* | 7 | 17 | 29 |  |
| **G + LF** |  | | | |
| WU2 | 24 | 0 | 100 | 0.1486 |
| WU2Δ*pspA* | 22 | 2 | 92 |  |

| **72 Hours** | **Recovered** | **Unrecoverable** | **% Recovered** | **P-value (Chi^2^)** |
| --- | --- | --- | --- | --- |
| **Untreated** |  | | | |
| WU2 | 8 | 20 | 29 | 0.0332 |
| WU2Δ*pspA* | 1 | 20 | 5 |  |
| **GAPDH** |  | | | |
| WU2 | 25 | 3 | 89 | ≤ 0.0001 |
| WU2Δ*pspA* | 5 | 16 | 24 |  |
| **Lactoferrin** |  | | | |
| WU2 | 7 | 17 | 29 | 0.0645 |
| WU2Δ*pspA* | 2 | 22 | 8 |  |
| **G + LF** |  | | | |
| WU2 | 22 | 2 | 92 | 0.0330 |
| WU2Δ*pspA* | 16 | 8 | 67 |  |
