# Supplementary material for: PspA-mediated aggregation protects Streptococcus pneumoniae against desiccation on fomites
Source: mBio. 2023 Nov 20;14(6):e02634-23. doi: 10.1128/mbio.02634-23 (PMC10746202; doi:10.1128/mbio.02634-23)
Supplement: Table S2 — Strains used (64). [file mbio.02634-23-s0003.docx]

**Table S2. Strains used.**

| **Strain** | **Serotype** | **Genotype** | **Reference** |
| --- | --- | --- | --- |
| WU2 | 3 | Clinical isolate | Hollingshead, Becker et al. 2000 (32) |
| WU2Δ*pspA* | 3 | Isogenic deletion mutant of full length gene of *pspA*, *ermR* | Park et al. 2021 (37) |
| D39 | 2 | Clinical isolate | Lanie et al. 2007 (63) |
| D39Δ*pspA* | 2 | Unmarked in-frame deletion of *pspA* gene by allelic-exchange using the Janus cassette | This study |
| TIGR4 | 4 | Clinical isolate | Tettelin et al. 2001 (60) |
| TIGR4Δ*pspA* | 4 | Unmarked in-frame deletion of *pspA* gene by allelic-exchange using the Janus cassette | This study |
| EF3030 | 19F | Clinical isolate | Mukerji et al. 2012 (35) |
| EF3030Δ*pspA* | 19F | Isogenic deletion mutant of full length gene of *pspA*, *ermR* | Park et al. 2021 (37) |
| MT15 |  | NEB®Express Iq  Competent *E. coli*/ pQE30-SS01 | Park et al. 2021 (38) |
| MT51 |  | BL21-DE3/pET30-2-GAPDH (human) | Park et al. 2021 (38) |
